# Inhibition of lung cancer cells and Ras/Raf/MEK/ERK signal transduction by ectonucleoside triphosphate phosphohydrolase-7 (ENTPD7)

**DOI:** 10.1186/s12931-019-1165-0

**Published:** 2019-08-23

**Authors:** Zhongwei Wen, Rongfang Jiang, Ying Huang, Zhineng Wen, Dong Rui, Xiaoxiao Liao, Zhougui Ling

**Affiliations:** Department of Respiratory and Critical Care Medicine, the Fourth Affiliated Hospital of Guangxi Medical University, No. 1 Liushi Road, Liuzhou, 545005 Guangxi Province China

**Keywords:** Lung cancer, Ectonucleoside triphosphate phosphohydrolase-7, Proliferation, Senescence

## Abstract

**Background:**

The aim of this study was to investigate the effects and mechanisms of ectonucleoside triphosphate phosphohydrolase-7 (ENTPD7) on lung cancer cells.

**Methods:**

The expression characteristics of ENTPD7 and its effect on the survival of lung cancer patients were analyzed by referring to The Cancer Genome Atlas (TCGA). Streptavidin-peroxidase (SP) staining was performed to detect the ENTPD7 protein in tumor tissues and adjacent tissues. Plasmid transfection technology was also applied to silence ENTPD7 gene. Crystal violet staining and flow cytometry were performed to determine cell proliferation and apoptosis. Tumor-bearing nude mice model was established to investigate the effect of sh-ENTPD7 on tumors.

**Results:**

The results showed that patients with low levels of ENTPD7 had higher survival rates. ENTPD7 was up-regulated in lung cancer tissues and cells. Down-regulation of the expression of ENTPD7 inhibited proliferation but promoted apoptosis of lung cancer cell. Silencing ENTPD7 also inhibited the expression levels of Ras and Raf proteins and the phosphorylation of mitogen-activated protein kinase (MEK) and extracellular signal-regulated kinase (ERK). Tumor-bearing nude mice experiments showed that silencing ENTPD7 had an inhibitory effect on lung cancer cells.

**Conclusions:**

ENTPD7 was overexpressed in lung cancer cells. Down-regulating ENTPD7 could inhibit lung cancer cell proliferation and promote apoptosis via inhibiting the Ras/Raf/MEK/ERK pathway.

## Background

Lung cancer is one of the most lethal malignant tumors. According to statistics from the American Cancer Society, lung cancer had the second highest incidence and the highest mortality rate in the United States in 2017 [1, 2]. Lung cancer is divided into small cell lung cancer (SCLC) and non-small cell lung cancer (NSCLC), with NSCLC accounting for about 80% of all cases [3]. Surgery, radiotherapy, chemotherapy and immunotherapy are the main methods for treating lung cancer, though the outcome of those methods is quite different [4]. Infiltration and distant metastasis in situ often occur to patients with lung cancer, and the 5-year survival rate of patients is still low [5–7]. Moreover, although tyrosine kinase inhibitor (TKI) was well developed in recent years, the problem of high recurrence rate still exists [8].

The activation of tyrosine kinase receptor-associated signaling system plays an important role in the development and progression of lung cancer. Ras/Raf/mitogen-activated protein kinase (MEK)/extracellular signal-regulated kinase (ERK) regulates a variety of cellular processes including proliferation and apoptosis [9, 10]. Recent studies have found that Ras/Raf/MEK/ERK pathway also plays a critical role in regulating cellular senescence [11–13].

Cellular senescence is a state of cell cycle arrest initiated by various stimuli such as telomere shortening, oncogenes, or chemotherapies, and senescence can occur after cell proliferation (replication of senescence) or immediately after acute stress [14, 15]. Ecto-nucleoside triphosphate diphosphohydrolase (ENTPDase) family consists of eight members that regulate extracellular Adenosine Triphosphate (ATP) level and participates in cellular activities [16]. Study found that ectonucleoside triphosphate phosphohydrolase-7 (ENTPD7) played an important role in inhibiting cellular senescence in liver cancer [17]. However, to the best of our knowledge, no research has been conducted on the role of ENTPD7 in lung cancer.

Therefore, this study mainly explored the expression characteristics of ENTPD7 in lung cancer, the effects of ENTPD7 on the proliferation and apoptosis of lung cancer cells and the mechanism of action. Our study provides a new understanding on the treatment of lung cancer.

## Methods

### Bioinformatics analysis

The mRNA expression level of *ENTPD7* and different tumor node metastasis (TNM) stages were downloaded from The Cancer Genome Atlas (TCGA) database. The mRNA expression levels of *ENTPD7* in different lung cancer stages were determined using TCGA, and weighted gene co-expression network analysis (WGCNA) was applied to conduct correlation analysis of the expressions of *ENTPD7* and survival rate.

### Patients and samples

From July 2015 to July 2016, lung cancer tissues and adjacent tissues were collected from 24 males and 20 females (aged from 44 to 74 years old, with an average age of 53.89 ± 4.01 years old). All patients enrolled were diagnosed with lung cancer by pathology and did not have other malignancies. The patients were diagnosed for the first time and did not receive radiotherapy, chemotherapy or immunotherapy. The tissues were stored at − 80 °C prior to subsequent experiments. All human studies were approved by the Ethics Committee of Fourth Affiliated Hospital of Guangxi Medical University.

### Streptavidin-peroxidase (SP) staining

SP staining (Bioss, USA) was performed to detect the ENTPD7 protein levels in lung tissues and adjacent tissues. Specimens were cut into 4-μm-thick section and deparaffinezed in xylene. 0.01 mol/L citrate buffer solution was used for antigen retrieval and 50 μL peroxidase blocking solution was added to block endogenous peroxidase activity. The primary antibody was added according to the instructions and incubated at 4 °C for 12 h. The secondary antibody was added and incubated at room temperature for 10 min. Next, 100 μL DAB was added and held for 5 min, hematoxylin was used for counterstaining, and the staining was observed under a microscope.

### Cell culture

Human normal lung epithelial cell line (BEAS2B), lung adenocarcinoma cell line (A549), lung squamous cell carcinoma cell line (SK-MES-1), bronchioloalveolar carcinoma cell line (NCI-H1650), lung large cell cancer cell line (NCI-H1299) and lung giant cell cancer cell line (95-D) were purchased from American Type Culture Collection (Manassas, VA, USA) and cultured in RPMI 1640 or DMEM medium containing 10% fetal bovine serum (FBS) at 37 °C in an incubator (Gibco, USA) with 5% CO_2_.

### Cell transfection and grouping

Plasmid transfection technology was applied to silence *ENTPD7* gene, and the pGPU6/GFP/Neo plasmid vector containing small hairpin (sh)RNA targeting *ENTPD7* was purchased from Origene (USA). Plasmid-shRNA-transfection of cells was performed using Lipofectamine 2000 (Invitrogen, CA) according to the instructions. Empty vector was used as negative control (NC) group. To explore the effect of *ENTPD7* silencing on lung cancer cells, the cells were divided into control group (no transfection), vector group (cells transfected with pGPU6/GFP/Neo-NC-shRNA) and sh-ENTPD7 group (cells transfected with pGPU6/GFP/Neo-ENTPD7-shRNA).

### Cell counting kit-8 (CCK-8) assay

CCK-8 assay (Tongren, Japan) was performed to test the cell viability. Transfected cells (100 mL, 3 × 10^3^ cells/well) were inoculated in a 96-well plate and incubated at 37 °C with 5% CO_2_ for 24, 48 and 72 h. CCK-8 reagent was then added into each well and cultured together at 37 °C with 5% CO_2_ for 4 h. Optical density (OD) values at 450 nm were measured (ELX 800, Bio-Teck, USA).

### Crystal violet staining

The crystal violet staining (Baomanbio, China) was used to test the cell proliferation ability.

### Flow cytometry

Flow cytometry was used to detect cell apoptosis and the kits were purchased from BD Pharmingen (USA). 1 × 10^6^ cells were washed with PBS at 4 °C and re-suspended to a concentration of 4 × 10^5^ cells/mL. Propidium iodide (PI) and AnnexinV-FITC were added according to the instructions, and flow cytometer (FACSCalibur, Becton-Dickinson, USA) was used to detect the apoptosis rate.

### Mice modeling and sample collection

BALB/c mice (6–8 weeks, 22 g–26 g, SFP) were purchased from Laboratory Animal Center (China). Modeling and follow-up experimental programs had been approved by China Council on Animal Care. Twelve mice were randomly divided into 3 groups, which were control group (*n* = 4), vector group (*n* = 4) and sh-ENTPD7 group (*n* = 4). The A549 or SM-MES-1 cells (1 × 10^6^ each mouse) were subcutaneously injected into the back of the mice, which were normally fed for 15 days. Ten μg empty plasmid or 10 μg sh-ENTPD7 plasmid was dissolved in saline and injected via the tail vein. Totally 5 injections should be carried out, and each injection was performed every 3 days for 15 days. The protein expression of ENTPD7 in different mice model groups were determined by Western blot. The tumors were surgically removed and measured.

### Western blot

Proteins were determined using Western blot. Cells were lysed and supernatant was collected by centrifuging at 12000 rpm at 4 °C for 15 min. BCA assay was used to determine the protein concentration. SDS-PAGE gel was used in electrophoresis. PVDF membrane (Bio-Rad, USA) was transferred by a Trans-Blot Transfer Slot (Bio-Rad, USA) and blocked with 5% fat-free milk for 2 h at room temperature. Primary antibodies (anti-ENTPD7, Abcam, ab236644, dilution: 1:700; anti-BAX, Abcam, ab32503, dilution: 1:700; anti-BCL2, Abcam, ab692, dilution: 1:800; anti-CDKN2A, Abcam, ab51243, dilution: 1:800; anti-CIP1, Abcam, ab109199, dilution: 1:900; anti-P53, Abcam, ab26, dilution: 1:800; anti-MKI67, Abcam, ab92742, dilution: 1:800; anti-Ras, Abcam, ab52939, dilution: 1:600; anti-Raf-1, Abcam, ab137435, dilution: 1:700; anti-MEK, Abcam, ab178876, dilution: 1:700; anti-p-MEK, Abcam, ab194754, dilution: 1:700; anti-ERK, Abcam, ab54230, dilution: 1:800; anti-p-ERK, Abcam, ab65142, dilution: 1:800; anti-GAPDH, Abcam, ab8245, 36 kDa, dilution: 1:800) were added according to the instruction, and the samples were shaken at room temperature for 2 h and incubated at 4 °C for 12 h. Secondary antibodies (mouse anti-human IgG, Abcam, ab1927, dilution: 1:10000; rabbit anti-human IgG, Abcam, ab6759, dilution: 1:8000; rabbit anti-goat IgG, Abcam, ab6741, dilution: 1:10000; donkey anti-rabbit IgG, R&D, NL004, 1:5000; goat anti-mouse IgG, Abcam, ab6785, 1:8000;) were added and incubated at room temperature for 1.5 h. Chemiluminescence detection was carried out using ECL reagent (Huiying, Shanghai, China). All protein assays were performed in triplicate.

### Quantitative real-time polymerase chain reaction (qRT-PCR)

The expression levels of mRNA were determined by qRT-PCR. The cells were triturated and lysed, and RNA from the cells was extracted by RNA extraction kit (Promega, Beijing, China). Reverse transcription kit (TaKaRa, Japan) was used to synthesize cDNA. Reverse transcription reaction condition was set at 37 °C for 15 min, while reverse transcriptase inactivation condition was set at 85 °C for 15 s. qRT-PCR was performed with the qRT-PCR kit (TaKaRa, Japan). PCR was performed by activating DNA polymerase at 95 °C for 5 min, followed by 40 cycles of two-step PCR (at 95 °C for 10 s and at 60 °C for 30 s) and a final extension at 75 °C for 10 min and held at 4 °C. RNase-free water was used as the templates of negative control. All primers were obtained from Genewiz (Suzhou, Jiangsu China) and listed in Table 1. 2^*-ΔΔCT*^ was used to analyze the mRNA expression level, and the data were represented by ΔΔCt. All PCR reactions were performed in triplicate. GAPDH was used as an internal control, and the mRNA expression of each gene was normalized against GAPDH expression.

**Table 1:** The sequences of primers

Primer name	Sequence (5′-3′)	Product size (bp)
*ENTPD7*-forward	CCCCTTTACATCCTCTGCAC	
*ENTPD7*-reverse	GTCAAACTCCAACGGCAAAT	242
*BAX*-Forward	TCCACCAAGAAGCTGAGCGAG	
*BAX*-Reverse	TTCTTTGAGTTCGGTGGGGTC	188
*BCL2*-Forward	CTGGTGGACAACATCGC	
*BCL2*-Reverse	GGAGAAATCAAACAGAGGC	164
*CDKN2A*-Forward	GTGCTCACTCCAGAAAACTC	
*CDKN2A* -Reverse	AATGTCCTGCCTTTTAACGTAG	147
*CIP1*-Forward	AGTATGCCGTCGTCTGTTCG	
*CIP1*-Reverse	CTTGTCCCCCTCCCAGGTCA	178
*P53*-Forward	CTGAGGTCGGCTCCGACTATACCACTATCC	
*P53*-Reverse	CTGATTCAGCTCTCGGAACATCTCGAAGCG	260
*MKI67*-Forward	GCAGGACTTCACTTGCTTCC	
*MKI67*-Reverse	TCATTTGCGTTTGTTTCACG	144
*GAPDH*-Forward	CCATCTTCCAGGAGCGAGAT	
*GAPDH*-Reverse	TGCTGATGATCTTGAGGCTG	222

### Statistical analysis

All the experimental data were presented as mean ± standard deviation (SD). Statistical analysis was performed by SPSS 20 (SPSS, Inc., Chicago, IL, USA). Differences among the experimental groups were analyzed by One-way analysis of variance (ANOVA) followed by Turkey’s multiple comparison. Statistical significance was defined as *P <* 0.05.

## Results

### ENTPD7 was overexpressed in lung cancer

**Fig. 1:**
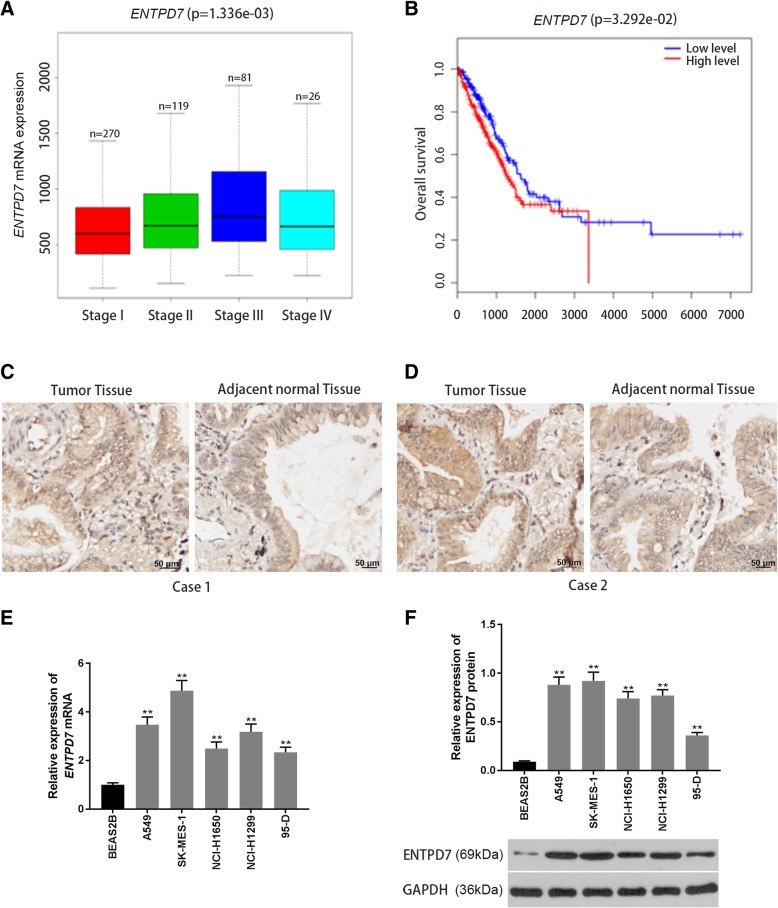
Expression characteristics of ectonucleoside triphosphate phosphohydrolase-7 (ENTPD7) (**a**) The Cancer Genome Atlas (TCGA) was used to determine the differential mRNA expression of ENTPD7 in patients at different lung cancer TNM stages. **b** Comparison of survival rates of patients at different ENTPD7 expression levels. **c**, **d** Streptavidin-perosidase (SP) staining was performed to detect ENTPD7 protein in lung tissues (*n* = 44) and adjacent tissues (*n* = 44). **e**, **f** Quantitative real-time polymerase chain reaction (qRT-PCR) and Western blot were performed to determine ENTPD7 mRNA and protein expression levels in different lung cells lines. All PCR reactions and protein assays were performed in triplicate. GAPDH was used as an internal control. ^*^P < 0.05, ^**^P < 0.01, versus BEAS2B group

A total of 496 cases (270 cases of stage I, 119 cases of stage II patients, 81 cases of stage III patients, and 26 cases of stage IV) of lung cancer were downloaded from the TCGA. The analysis showed that the mRNA expression levels of *ENTPD7* were different in tumor tissues of patients at different TNM stages (Fig. 1a). Further survival analysis also showed that patients with a low level of *ENTPD7* had a higher survival rate than patients with a high *ENTPD7* level (Fig. 1b). SP staining results also showed that *ENTPD7* had a higher expression level in lung cancer tissues than that in adjacent tissues (Fig. 1c, d).

The expression level of ENTPD7 in different lung cancer cell lines was detected by qRT-PCR and Western blot. The results showed that the expression levels of ENTPD7 mRNA and protein in A549, SK-MES-1, NCI-H1650, NCI-H1299 and 95-D cell lines were significantly higher than those in human normal lung epithelial cell line BEAS2B, and among them, ENTPD7 expression was the highest in A549 and SK-MES-1 cells (Fig. 1e, f). This suggested that the ENTPD7 gene was up-regulated in lung cancer tissues and cells.

### Effects of ENTPD7 silencing on A549 cells and SK-MES-1 cells

**Fig. 2:**
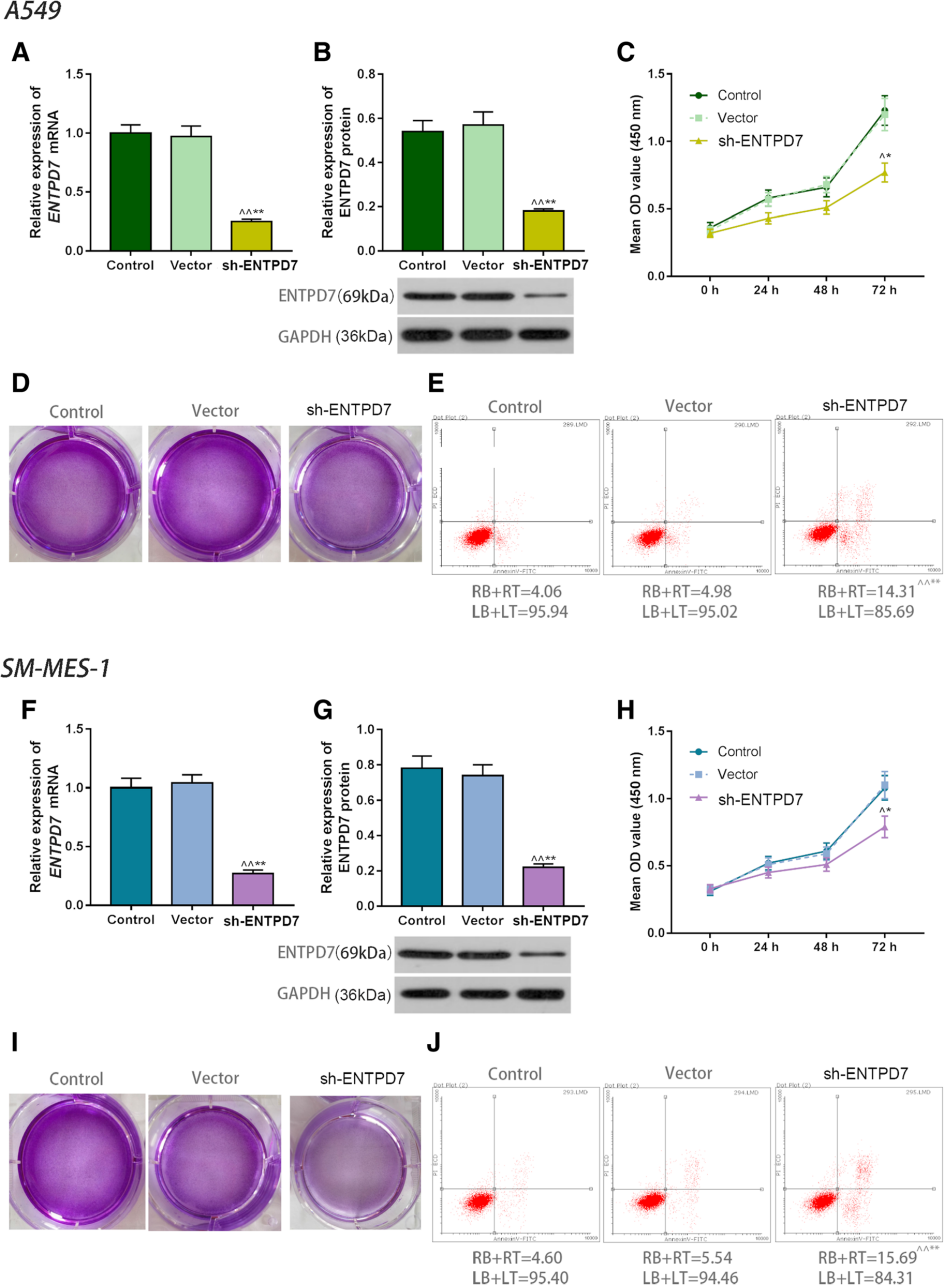
Effects of ectonucleoside triphosphate phosphohydrolase-7 (ENTPD7) silencing on A549 and SM-MES-1 cells. **a**, **b**, **f**, **g** ENTPD7 mRNA and protein expression levels were detected by quantitative real-time polymerase chain reaction (qRT-PCR) and Western blot. GAPDH was used as an internal control. All PCR reactions and protein assays were performed in triplicate. **c**, **h** Cell counting kit-8 (CCK-8) assay was performed to tested cell viability. **d**, **i** The crystal violet staining was used to test the cell proliferation ability. Pale violet staining represented a low proliferation, and dark violet staining meant high proliferation. **e**, **j** Apoptosis rates were detected using flow cytometry. A549 and SM-MES-1 cells were stained with Annexin V-FITC and PI. ^^^*P* < 0.05, ^^^^*P* < 0.01, versus control group; ^*^P < 0.05, ^**^P < 0.01, versus vector group

Western blot and qRT-PCR results showed that ENTPD7 protein and mRNA levels in the sh-ENTPD7 group were significantly lower than those in the control group, vector group of A549 cells and SM-MES-1 cells (Fig. 2a, b, f, g), suggesting that plasmid transfection successfully down-regulated the expression of ENTPD7. OD value in the sh-ENTPD7 group was greatly lower than that in the control group and vector group, while apoptotic rate in sh-ENTPD7 group was noticeably higher than that in the control group and vector group (Fig. 2c-e, h-j). This indicated that a low expression of ENTPD7 inhibited the proliferation but promoted apoptosis of A549 cells and SM-MES-1 cells.

### Effects of ENTPD7 silencing on proliferation and apoptosis

**Fig. 3:**
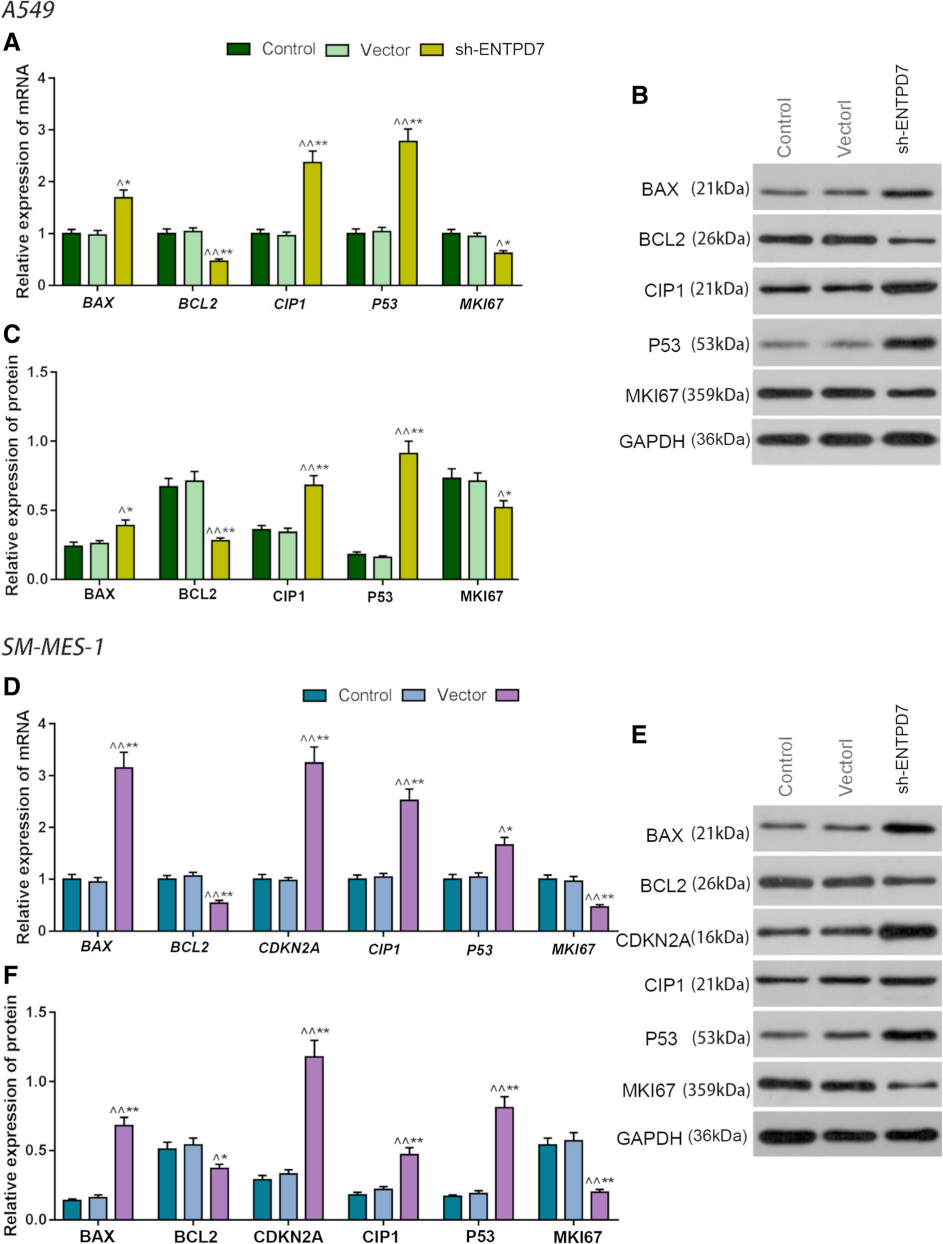
Effects of ectonucleoside triphosphate phosphohydrolase-7 (ENTPD7) silencing on proliferation and apoptosis. **a-f** Quantitative real-time polymerase chain reaction (qRT-PCR) and Western blot were performed to detect the protein and mRNAs levels of BAX, BCL2, CDKN2A, CIP1, P53 and MKI67. All PCR reactions and protein assays were performed in triplicate. GAPDH was used as an internal control. ^^^*P* < 0.05, ^^^^*P* < 0.01, versus control group; ^*^P < 0.05, ^**^P < 0.01, versus vector group

To further investigate the effects of ENTPD7 on proliferation and apoptosis, the levels of cell cycle-associated and apoptosis-related proteins in the three groups of two cell lines were determined. The results showed that the protein expression levels of BAX, CIP1 and P53 were up-regulated in the sh-ENTPD7 group of A549 cells and SM-MES-1 cells, the expression level of CDKN2A was also increased in the sh-ENTPD7 group of SM-MES-1 cells, however, the expression levels of BCL2 and MKI67 levels were down-regulated in the sh-ENTPD7 group (Fig. 3a-f). Our data demonstrated that down-regulation of ENTPD7 regulated the proliferation and apoptosis of lung cancer cells by modulating cell cycle-associated and apoptosis-related proteins.

### Effects of ENTPD7 silencing on Ras/Raf/MEK/ERK pathway

**Fig. 4:**
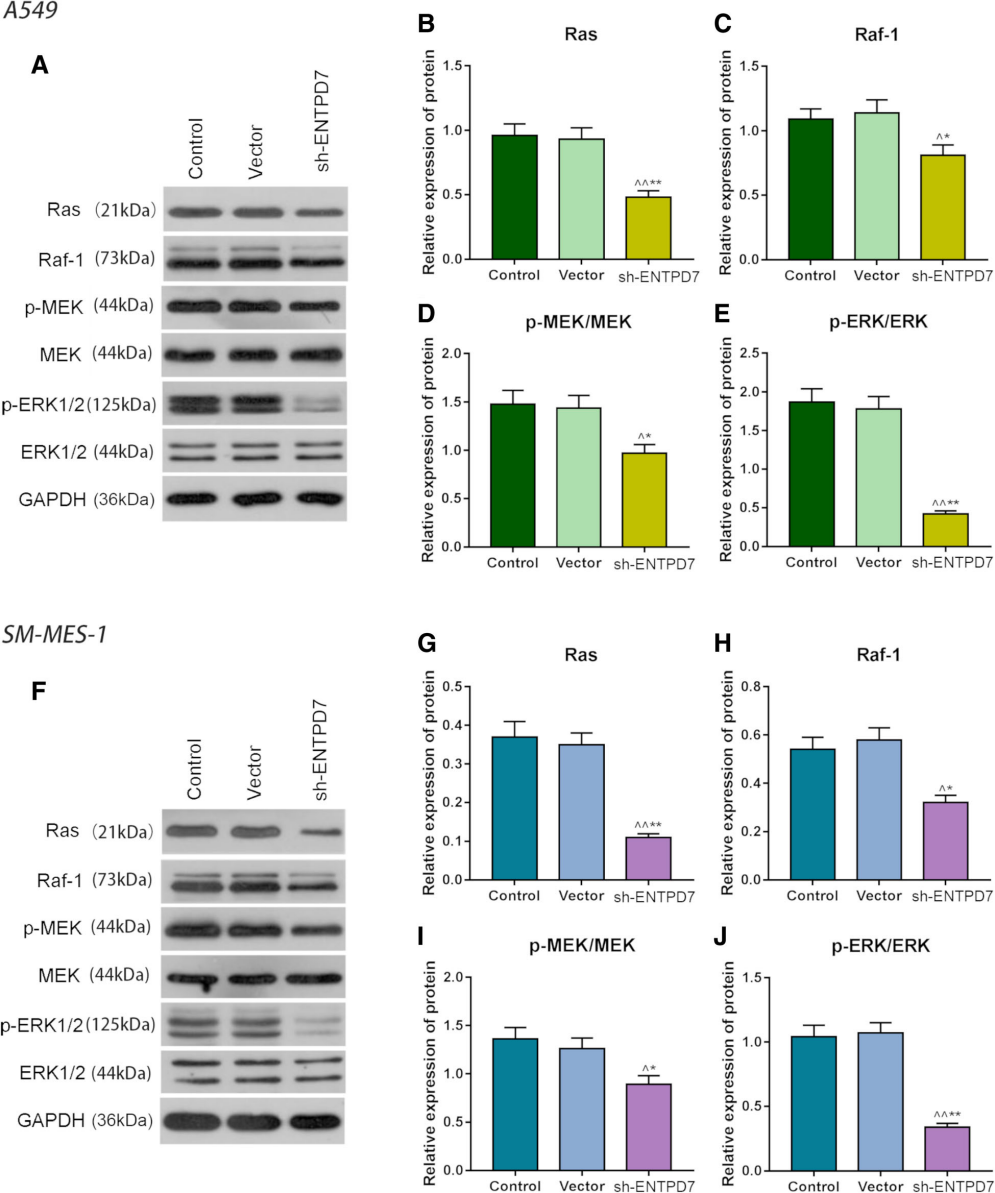
Effects of ENTPD7 silencing on Ras/Raf/MEK/ERK pathway. **a-j** Western blot was used to detect Ras and Raf proteins levels and phosphorylation levels of MEK and ERK protein in A549 and SM-MES-1 cells. All PCR reactions and protein assays were performed in triplicate. GAPDH was used as an internal control. ^^^*P* < 0.05, ^^^^*P* < 0.01, versus control group; ^*^P < 0.05, ^**^P < 0.01, versus vector group

To explore the mechanism by which ENTPD7 affects cell proliferation and apoptosis, the expression levels of proteins in the Ras/Raf/MEK/ERK pathway in the three groups of two cell lines were detected. The results show that in both cell lines, Ras, Raf protein expression levels and phosphorylation levels of MEK and ERK proteins in the sh-ENTPD7 group were significantly lower than those in the control group and vector group (Fig. 4a-j). This suggested that ENTPD7 with a low expression inhibited Ras/Raf/MEK/ERK pathway.

### Effects of ENTPD7 silencing on lung tumor in vivo

**Fig. 5:**
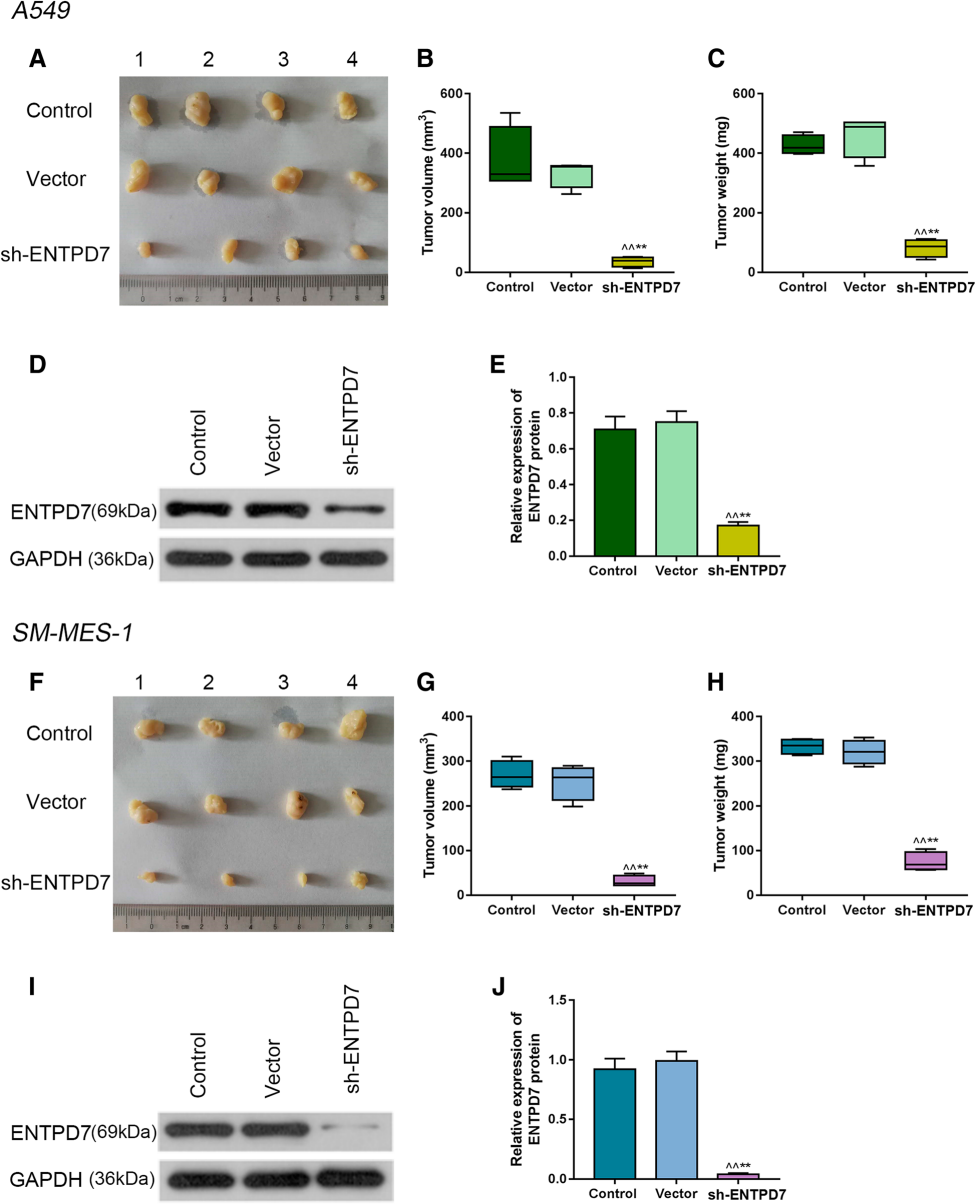
Effects of ectonucleoside triphosphate phosphohydrolase-7 (ENTPD7) silencing on lung tumor in vivo. **a-c**, **f-h** The volume and quality of subcutaneous tumors in tumor-bearing nude mice were compared. **d-e**, **i-j** Western blot was used to detect ENTPD7 proteins levels in tumor-bearing nude mice. All experiments were performed in triplicate. ^^^*P* < 0.05, ^^^^*P* < 0.01, versus control group; ^*^P < 0.05, ^**^P < 0.01, versus vector group

The results showed that the expression level of ENTPD7 in sh-ENTPD7 group was significantly lower than that in the control group and vector group, and the tumor volume and weight in the sh-ENTPD7 group were greatly smaller than those in the control group and vector group (Fig. 5a-j), showing that the sh-ENTPD7 plasmid had an inhibitory effect on lung tumor.

## Discussion

Nucleotides have different functions in cells. Studies have shown that nucleotides play an important role in cell metabolism, proliferation and apoptosis, thus, the level of ATP is tightly regulated [18–20]. The levels of extracytoplasmic (extracellular and intraorganellar) nucleotides are modulated by the degradative activity of ENTPDs [21, 22]. Research has found that ENTPDs were also associated with cancer, as ENTPD5 affected the proliferation of liver cancer cells [23]. Moreover, by conducting bioinformatics research, we found that ENTPD7 levels in lung cancer patients at different TNM stages were different, and the survival analysis showed that lung cancer patients with a low level of ENTPD7 had a higher survival rate. Therefore, ENTPD7 was selected to be used in the further research.

We first determined the expression characteristics of ENTPD7 in lung cancer tissues by using clinical samples. SP staining results showed that the expression level of ENTPD7 in lung cancer tissues was higher than that in adjacent tissues. Furthermore, the results also showed that the mRNA and protein levels of ENTPD7 in the five lung cancer cell lines were significantly higher than those in normal lung epithelial cells, showing that ENTPD7 was overexpressed in lung cancer tissues and cells. To further investigate the effects of ENTPD7 gene on lung cancer cells, A549 and SK-MES-1 cells with high ENTPD7 expression were selected as subjects, and ENTPD7 was silenced by plasmid transfection. The results showed that the down-regulation of ENTPD7 expression inhibited the proliferation but promoted apoptosis of A549 and SK-MES-1 cells.

In order to study the mechanism underlying the inhibition of ENTPD7 on lung cancer cells, we reviewed previous research, though only a few papers focused on the direct effect of ENTPD7 on tumor cell apoptosis or proliferation, ENTPD7, as an enzyme that regulates nucleotide levels, was shown to have a function of modulating ATP, which in turn regulates the transduction of signals for phosphorylation of proteins in signaling pathways [24, 25].

Abnormality of cell proliferation or apoptosis caused by mutation or activation of receptor tyrosine kinase (RTK) and its related pathways is one of the characteristics of tumors [26]. The up-regulation of Ras/Raf/MEK/ERK pathway promotes cell proliferation, and inhibits apoptosis [27, 28] and cell senescence [29–31]. Studies have shown that down-regulation of Ras/Raf/MEK/ERK and p16INK4A/Rb signaling attenuates tumor cell senescence induced by baicalin [32]. Peng [33] also showed that in NSCLC, the inhibition of senescence caused by Raf mutation is involved in the development of lung cancer. Cellular senescence, which refers to the irreversible cell cycle arrest of cells, can inhibit the proliferation of cells, thus, inducing cell senescence is an important method in tumor therapy [34]. Replicative senescence (RS) caused by telomere shortening [35], oncogene-induced senescence (OIS) caused by oncogenes activation such as RAS or B-Raf^V600E^ [36], PTEN-loss induced cellular senescence (PICS) [37] and stress-induced senescence [38] are currently triggered cell senescence. After the senescence signal is triggered, senescence-related proteins such as p16, p21 and p53 are activated and could cause cell senescence [39, 40]. p53-p21 and p16-Rb tumor suppressor pathways are pathways for cellular senescence, and Ras-induced senescent cells are often associated with the up-regulation of p16, p53 and p21 [41, 42]. It is currently believed that Ras mainly relies on the RAF/MEK/ERK pathway in regulating key proteins such as p14/p19, p53 and p16 expression and causing cell senescence [43]. Once Ras is activated, the downstream RAF is recruited to the cell membrane and activated. RAF subsequently phosphorylates MEK, which phosphorylates and activates the extracellular regulatory protein to over-activate ERK1/2, therefore leading to the accumulation of intracellular oxygen free radicals, causing DNA damage response and cell senescence [44–46].

The results of this study showed that silencing ENTPD7 up-regulated the levels of CDKN2A, CIP1 and P53 but down-regulated the expression level of MKI67. P16 (CDKN2A), p21 (CIP1) and p53 (P53) are not only the main senescence -associated proteins, but also important markers of cell cycle. Tordella et al. found that ENTPD7 expression induced several senescent effectors in liver cancer cell including p16INK4a, p53 and p21 [17]. Same gene showing different mechanisms in different tumor may be related to the type of tumor cells, living environment or other factors. Tumors are heterogeneous populations of cells, and the intra-tumor heterogeneity further enhances the effect of clone variation and microenvironment on cancer cells [47]. Heterogeneity of tumor and microenvironment allow a gene to have different functions and effects on different tumor cell lines, however, such a functional difference still needs to be further confirmed. As a tumor suppressor gene, p53 could inhibit cell proliferation by arresting the cell cycle [48], while p21 gene act as a cyclin-dependent kinase inhibitor downstream of p53 gene [49]. P21 can form a cell cycle G1 checkpoint with p53 [50]. Ki67 (MKI67) is a non-histone in the nucleus and is expressed in every cell cycle. However, when M phase is completed, Ki67 will degrade rapidly, thus, cells in the G0 phase do not express ki67 [51, 52]. In addition, the results also showed that the protein expression levels of Ras, Raf and phosphorylation levels of MEK and ERK proteins were significantly down-regulated after down-regulating ENTPD7, suggesting that ENTPD7 could promote the expressions of p21 and p53 by inhibiting the expression and activation of Ras/Raf/MEK/ERK pathway, thereby inducing cell senescence, arresting cells in G0 phase and finally inhibiting cell proliferation. In addition, Ras/Raf/MEK/ERK pathway also has an effect of promoting apoptosis, and the result experiment also showed that inhibiting ENTPD7 could promote the expression of pro-apoptotic protein Bax and inhibit the level of anti-apoptotic protein Bcl-2 [53, 54]. This demonstrated that the inhibition of ENTPD7 might inhibit lung cancer cell proliferation and promote apoptosis by down-regulating protein expression and phosphorylation of the protein in the Ras/Raf/MEK/ERK pathway. Further animal experiments in vivo have also shown that the intravenous administration of sh-ENTPD7 plasmid had an effect of inhibiting the growth of lung cancer cells.

## Conclusions

In conclusion, ENTPD7 was overexpressed in lung cancer tissues and cells, and the inhibition of ENTPD7 had the effects of inhibiting lung cancer cell proliferation and promoting apoptosis of lung cancer cells, and this might be related to the inhibition of Ras/Raf/MEK/ERK induced by ENTPD7.

## Data Availability

The analyzed data sets generated during the study are available from the corresponding author on reasonable request.

## Abbreviations

ANOVA: Analysis of variance
ENTPD7: Ectonucleoside triphosphate phosphohydrolase-7
ENTPDase: Ecto-nucleoside triphosphate diphosphohydrolase
ERK: Extracellular signal-regulated kinase
MEK: Mitogen-activated protein kinase
NSCLC: Non-small cell lung cancer
OD: Optical density
OIS: Oncogene-induced senescence
PICS: PTEN-loss induced cellular senescence
RS: Replicative senescence
RTK: Receptor tyrosine kinase
SCLC: Small cell lung cancer
SD: Standard deviation
SP: Streptavidin-peroxidase
TCGA: The Cancer Genome Atlas
TKI: Tyrosine kinase inhibitors
TNM: Tumor node metastasis
WGCNA: Weighted gene co-expression network analysis
